# Tympanoplasty Outcomes: A Review of 789 Cases

**Published:** 2015-03

**Authors:** Shabbir Indorewala, Taiwo Olugbemiga Adedeji, Abuzar Indorewala, Gaurav Nemade

**Affiliations:** 1 *DNB Institution and Research, Indorewala ENT Hospital, Nashik, India.*; 2 *Department of Otorhinolaryngology Head and Neck Surgery, LAUTECH Teaching Hospital, Osogbo, Nigeria. *

**Keywords:** Endomeatal tympanoplasty, Hearing improvement, Graft materials, Tympanoplasty

## Abstract

**Introduction:**

Tympanoplasty is indicated to restore hearing disability and prevent recurrent otorrhea.

**Materials and Methods:**

This study was a retrospective review of patients who underwent tympanoplasty with or without mastoidectomy over a 1-year period.

**Results:**

A total of 789 tympanoplasties were reviewed, with a male-to-female ratio of 1:1.1. In total, 91% and 9% of tympanoplasties were performed without and with mastoidectomy, respectively. Complete graft take was observed in 98.6% of cases. Approximately 25% of patients had an air-bone gap (ABG) gap ≤20dB pre-operatively, increasing to 75.6% post-operatively. ABG closure improved from 0.8% to 46.7%. Mean ABG improved from 26.30 ±8.1dB pre-operatively to 14 ± 10.41dB post- operatively (t=28.7, P<0.001). Generally, over 86% of patients had improvement in their hearing function post-operatively (mean= 12.5 ±9.5dB) (χ2= 104.2, P<0.001).

**Conclusion:**

Tympanoplasty is an effective procedure that can lead to improvement in hearing function in patients and prevention of recurrent ear discharge. Optimal results can be achieved through use of the appropriate surgical technique.

## Introduction

Chronic suppurative otitis media (CSOM) constitutes a major public health problem in children and adults in the developing world (1,2). It is an infection characterized by recurrent middle-ear discharge through a persistent tympanic membrane perforation. The disease is the most common childhood infectious disease worldwide, starting early in life (1). However, in the developing world, risk factors such as malnutrition, over-crowding, substandard hygiene, frequent upper respiratory tract infections, and under-resourced healthcare compound the problems and make the disease prevalent among children and adults (1,3–6). 

A consequence of CSOM is hearing loss and a propensity to recurrent infection and discharge (4-6). Adoga et al. (1) stated that of all the complications associated with CSOM, hearing loss is nearly always significant. Prevalence of hearing loss complicating CSOM ranges from 9–83% have been reported (1,4,5). 

Surgical repair (tympanoplasty) of the perforated tympanic membrane (TM) is indicated to restore hearing ability as well as to prevent recurrent otorrhea (7). Tympanoplasty was introduced by Berthold and later developed and modified by Wullstein and Zollner (4,7-9). The various surgical approaches to tympanoplasty include endomeatal (per meatal), endaural, and post-auricular routes. These approaches have a different effect on surgical outcome, depending on the size and site of perforation (7). A surgical technique using either underlay or overlay of grafts over the perforated TM has been employed by various surgeons (7,10,11). The underlay is widely used and is relatively simple to perform, as the graft is placed entirely medial to the remaining drum and malleus (4,7,12).

Different TM reconstruction techniques for tympanoplasty using different types of grafts, including temporalis fascia, perichondrium, palisade cartilage and Cartilage Island, have been described (13–15). While temporalis fascia has better functional outcome with respect to hearing (13,15), it is subject to poor dimensional stability (16). The poor dimensional stability of temporalis fascia grafts contributes to residual perforations following tympano- plasty, particularly in large TM perforations (16). Palisade cartilage and cartilage island techniques have the disadvantage of interfering with the sound-conducting mechanism, and hence patients may benefit only minimally with regard hearing restoration (7). 

Use of fascia lata as a graft material was reported to have better dimensional stability and subsequently leads to better outcome, particularly with respect to achieving an intact TM and hearing improvement (16,17). In contrast, the anterior tympanotomy technique was introduced to overcome anterior blunting associated with the underlay technique and also to take care of residual anterior perforation (17). 

This study aims to evaluate the outcome of tympanoplasty based on closure of TM perforation and hearing improvement using the reconstruction techniques of facial lata graft and anterior tympanotomy in tympanoplasty procedures.

## Materials and Methods

This study was a 1-year retrospective review of all patients undergoing middle-ear reconstructive surgery (tympanoplasty) with or without mastoidectomy at Indorewala Ear, Nose and Throat (ENT) Hospital, DNB Institution and Research, Nashik, India between January and December 2012. Data were obtained from the clinical records of patients who had been followed-up for a minimum of 6 months after surgery. Information retrieved by the investigator from the case record of patients included socio-demographics, the affected ear, site and size of the TM perforation, flap raised and graft materials used for reconstruction. Outcomes measured for closure of TM perforation were assessed as full take, partial take, or graft rejection 3 months after surgery, while the post-operative hearing of the patients was also assessed. A ‘full take’ was recorded when the TM had complete closure as seen under otomicroscopy, partial take was regarded as <100% closure, and graft failure was defined as complete rejection of the graft.

The pre- and post-operative pure tone audiometry (PTA) evaluations for all patients were performed using an Elkon 3N3 Multi (Elkon, India), calibrated yearly to International Organization for Standardization (ISO) standards in a sound-proofed room.Air conduction (AC) and bone conduction (BC) Pure tone average (PTA) values were calculated as the mean of the hearing thresholds at frequencies of 0.5, 1, 2, and 4 kHz. Air-bone gaps (ABG) were calculated as the difference between the AC-PTA and BC-PTA thresholds. Post-operative hearing gain was calculated as the difference between the ABG before and after follow-up examination (a minimum of 3 months after surgery when all healing processes had been judged complete and the patient was clinically stable). 

Patients with incomplete information and those lost to follow-up before healing was completed were excluded from the study. The data were entered into a spreadsheet and presented in a descriptive form as tables. A statistical analysis was performed using statistical package for social sciences (SPSS) version 14 (Chicago, IL) with means, frequencies calculated. The data were presented in simple descriptive terms as proportions using tables and graphic charts.

*Choice of graft material and its preparation*

Although the use of fascia lata as a graft material is not very common or popular, the choice of this graft material in our center was based on a previous study in which the dimensional stability of a fascia lata graft was compared with that of a temporalis fascia graft in both animal and humans (16,17). The fascia lata graft was found to have good dimensional stability compared with temporalis fascia in both animals and humans. This study concluded that the poor dimensional stability of temporalis fascia grafts can be an important contributor to the incomplete closure of TM perforations in tympanoplasty operations, particularly in large perforations.

*Preparation of the graft*

The graft was harvested from the anterolateral aspect of the distal third of the thigh. A small (0.5-mm) incision was made after infiltration of the area where the graft was to be harvested (using 1% lignocaine with 1 in 30,000 adrenaline). The fat was gently separated by blunt dissection to reveal the fascia lata, and a graft measuring approximately 24 mm by 16 mm was harvested. The fat and loose connective tissues from both sides of the fascia lata were then removed. Then all the four corners were cut by 8 mm in length and 4 mm in breadth to avoid a dog-ear formation. A 3-mm slit was made in the center at the base of the superior tail to accommodate the malleus handle. The graft was now ready for final placement. The incision was closed with a 2/0 nylon/silk suture and the dressing was put in place. The stitch was usually removed on the seventh post-operative day and the wound usually healed without any morbidity. Graft preparation is shown in (Fig. 1a-1c).

**Fig 1a:**
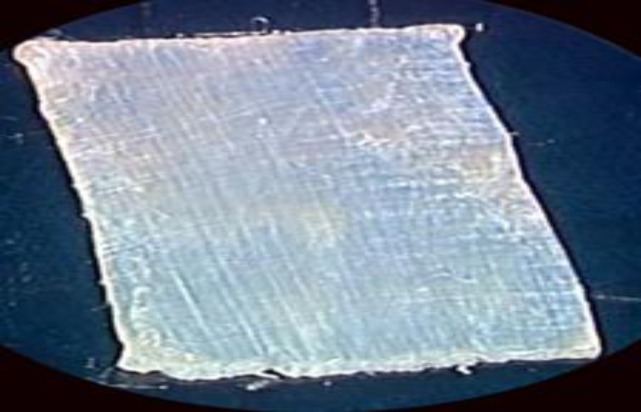
A 16mm*24mm fascia lata just before trimming the comers

**Fig1b:**
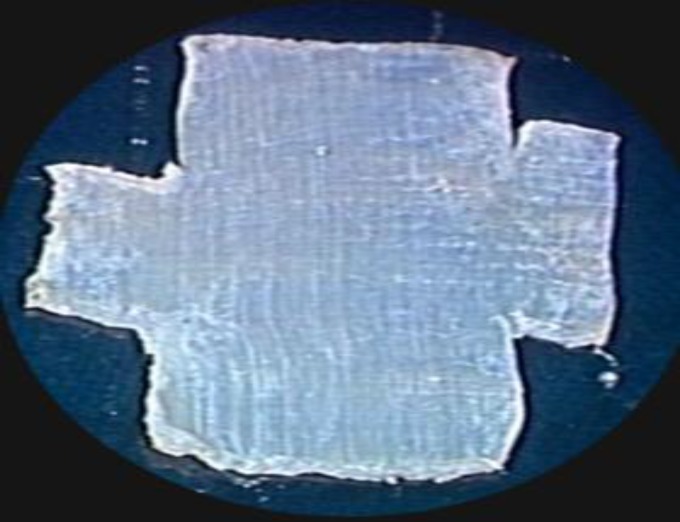
The four comers of the fascia are cut (each 4mm.8mm). The graft now has central piece and four tails. Cutting comers facilitate the final adaptation of the graft under the tympanic membrane and the tails tum on the canal walls. The dog-ear formation is avoided as the comers are removed

**Fig 1c:**
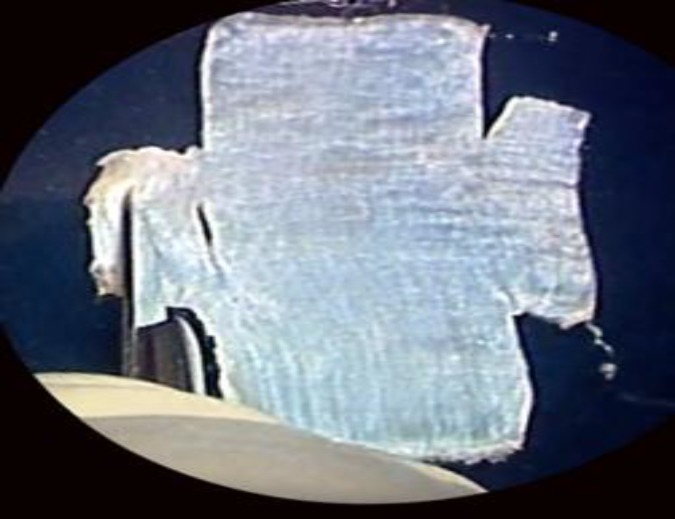
A slit is made at the base of the superior tail for malleus handle fishing

## Results

A total of 937 tympanoplasties (with or without mastoidectomies) were performed during this study. In total, 789 cases (from 637 patients, 485 patients had unilateral ear surgeries and 152 patients had bilateral ear surgeries) had complete data for analysis. There were 302 (48.72%) males and 335 (51.3%) females (male:female ratio, 1: 1.1). The age of the patients ranged from 5 to 76 years, with a mean age of 35±15.8 years; the age group 21–40 years was the most affected group (36%). Table. 1 shows the age, gender, and laterality of the affected ears and indications for surgery. 

**Table1:** Clinical characteristics of the patients

**Variable Age (years)**	**Frequency**	**Percentage**
1 – 15	80	12.6
15 – 30	189	29.6
31 – 45	212	33.3
46 – 60	113	17.7
61 and above	43	6.8
Mean ±SD	35 ± 15.79	
**Gender**		
Male	302	47.4
Female	335	52.6
**Affected ear**		
Right	384	48.7
Left	405	51.3
Total	789	100.0

TM perforations were small (<30%) in 226 cases (28.6%), medium/large (30–60%) in 255 cases (32.3%) and subtotal/total (>60%) in 308 cases (39.0%). There were central perforations in 658 (83.4%) ears and attic perforation with retraction pockets in 131 (16.6%) ears, and 162 (20.5%) cases had associated ossicular erosion ranging from erosion of the malleus handle to the absence of stapes superstructure (Table. 2).

**Table 2:** Pre-operative ossicular and audiometric findings

**Variable**	**Frequency**	**Percentage**
**Ossicular findings (N=162)**		
Resorption of malleus handle	25	15.5%
Necrosis of long process of incus	91	56.2%
Absence of stapes superstructure	35	21.6%
Absence of malleus, incus & stapes superstructure	11	6.8%
**Audiometric findings** Air-bone gap ABG range (dB HL)		
1.0 -10.0	14	1.8%
10.1-20.0	174	22.1%
20.1-30.0	382	48.4%
30.1-40.0	183	23.2%
40.1-50.0	34	4.3%
50.1-60.0	2	0.3%

A total of 718 (91.0%) tympanoplasties without mastoidectomy were performed via an endomeatal approach, while 71 (9.0%) were tympanoplasty with mastoidectomy via a post-auricular approach. Access to the middle ear was obtained by raising posterior and anterior tympanomeatal flaps in 408 (51.7%) patients with underlay graft placement technique in all patients. A fascia lata graft was used in 88.3%, temporalis fascia in 9.3% and tragal perichondrium in 2.4% of the patients. (Table. 3) shows the relationship of the graft materials and successful ABG closure achieved and access to the middle ear during reconstruction. Over 98% full graft take and complete closure of perforations was recorded. Pre-operative PTA showed that over 98% of patients had ABG >10 dB (mean, 26.3±8.1 dB) (Table. 2).

**Table 3:** Relationship of the graft materials and successful tympanic membrane closure achieved and various accesses to the middle ear during reconstruction.

**A. Graft materials**	**Outcome**			**Total**
	Complete closure	(75-99% )take	no take	
Facial lata graft	686 (98.4%)	10 (1.4%)	1 (0.2%)	697
Temporalis fascia	73 (100.0%)	0 (0.00%)	0	73
Tragal perichondrium	19 (100.0%)	0 (0.00%)	0	19
Total	778	10	1	789
**B. Flaps for access to the middle ear**				
**Flaps raised**	**frequencies**			**percentage**
Anterior + Posterior	642			81.4%
Posterior	147			18.6%
Total	789			100%

**Fig2:**
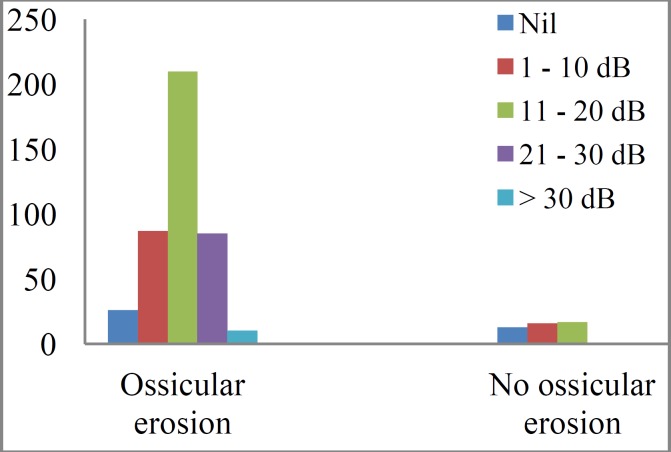
Comparison of air - bone gap closure according to the type of otological procedure

**Fig 3:**
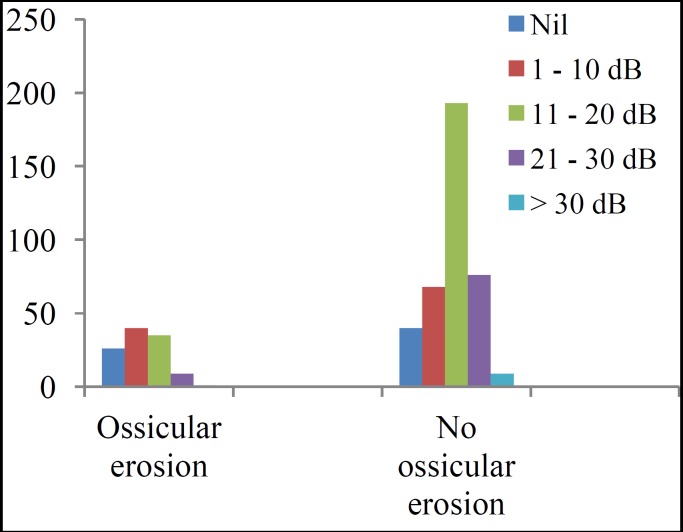
Air – bone gap closure achieved according to ossicular pathology

A total of 492 (62.4%) cases had PTA for post-operative hearing evaluation. Pre- and post-operative audiograms of these patients were compared for evaluation of post-operative ABG closure. While 25.2% of the patients had ABG ≤20dB before surgery, the proportion increased to 75.6% after surgery. Four patients (0.8%) had ABG <10dB pre-operatively, and the proportion increased to 46.7% post-operatively. The mean ABG was 26.3±8.1dB pre-operatively and 14.0±10.4 dB post-operatively (t=28.7, P<0.001). More than 86% of patients had objective improvement (shown by ABG closure) (χ^2^=104.2, P<0.001) post-operatively with a mean ABG closure of 12.5 ±9.5dB. Fig. 2 shows the relationship between outcome in relation to hearing and surgery type (tympanoplasty alone and tympanoplasty plus mastoidectomy; χ^2^=23.36, P<0.001), while Fig.3 demonstrates the relationship between outcome of surgery and ossicular erosion (χ^2^=33.28; P<0.001).

* X* ^2^= 23.36; P < 0.001

Chi Square = 33.28; P<0.001 23.36; P<0.001

## Discussion

The present study showed the relevance and importance of surgical closure (tympanoplasty) in the management of patients with TM perforation especially due to chronic middle-ear infections. The reason for the marginal female predominance found in this study is not known. While females have been found to pay more attention to their health, some other studies have reported a male preponderance (4,7). The fact that CSOM was the major indication (93.9%) for surgery in this study corroborates the assertion that this condition constitutes a major public health problem in both children and adults, especially in the developing world (1–6), and that surgical management is effective at preventing recurrent ear discharge and restoring hearing (7,10,13). 

CSOM as the major indication for tympanoplasty has also been published in previous studies (4,7,10-12,18). Other indications for tympanoplasty have been reported in literature (19-21). 

In the USA, however, Te et al. (19) reported iatrogenic perforations from ventilation tube therapy in 93 children to be the main indication for tympanoplasty in their study. 

The endomeatal approach was used in all our patients who had tympanoplasty without mastoidectomy. Using this route, the posterior and anterior tympanomeatal flaps (anterior and posterior tympanotomy) raised have been shown to be effective for disease clearance, proper graft placement, and better surgical outcome as well as ensuring stitchless surgery (17,22). Other patients with cholesteatoma were managed by tympanoplasty combined with modified radical mastoidectomy via the post-auricular approach. 

Although the type of graft material used for closure of TM perforations has been shown to have an effect on outcome of surgery, there are no consistent success rates for achieving an intact tympanic membrane after surgery using different surgical techniques (13,14,16). In Turkey, Kazikdas et al. (13) compared the use of palisade cartilage with the temporalis fascia tympanoplasty technique for the management of subtotal perforations and reported a better outcome with the palisade cartilage group. Sheehy et al. (23) compared the use of canal skin with that of temporalis fascia as a graft material for closure of TM perforations and reported that the graft take and hearing results were less favorable with the use of canal skin. Demirpehlivan et al. (14) compared different TM reconstruction techniques in type I tympanoplasty. They reported better outcome regarding closure of perforation with perichondrium/cartilage island than with either temporalis fascia or cartilage palisades. 

Use of fascia lata as graft material in tympanoplasty procedures has been studied in both animal and human experiments and, due to its dimensional stability (16,17), has proved to have better outcomes than the temporalis fascia. The findings of the present study, however, show that residual perforations occurred among patients whose repair was conducted with a facial lata graft. This may be due to the fact that a fascia lata graft was used in the majority (88.3%) of our patients. The residual perforations might also have resulted from cauterization of granulation tissue during the healing process. Another factor may be that most cases of large and total perforation were repaired with a fascia lata graft. The cause of the only case of graft failure was, however, not known. Generally, the outcome of surgery in this study was subjectively very good, with over 98% complete closure of perforation, comparable to 99% from previous study in this center but much higher than findings from many other previous published studies (4,10–15,18,19). The anterior tympanotomy technique has been reported to afford the surgeon an improved view of the aural end of the Eustachian tube, and thus allow complete removal of disease around its mouth (19). Furthermore, it provides an opportunity for firm anterior adaptation of the graft material which prevents post-operative blunting and its associated residual perforation (17,19). An anterior tympanotomy approach was employed in 81.4% of the patients in the present study, and this might have contributed to successful closure of TM perforation in the larger proportion of our patients.

Although there are controversies concerning the benefit of tympanoplasty in improving the functional hearing results in affected patients (24,25), published studies reported its benefit based on the subjective and objective improvement on patients’ hearing function (4,10-15). Sergi et al. (7) reported that tympanoplasty resulted in a 57–97% improvement in patients’ hearing function and that myringoplasty can improve hearing independent of the site and size of perforation, and thus concluded that hearing improvement can be used as an indication for myringoplasty. Mishra et al. (12) reported hearing gain of 10–30 dB in 95% of their cases. Faramarzi et al. (10) reported that approximately 24% patients that had ABG within 25 dB before intervention; increasing to 71% post-operatively. Demirpehlivan et al. (14) in their study on the comparison of different TM reconstruction techniques in type I tympanoplasty reported improvement in average PTA post-operatively, regardless of the materials used for the reconstruction. 

In this study, 86.6% of the patients had a gain in hearing function which is within the range of findings from other published study (7). Olusesi et al. (4) and Ogisi et al. in Nigeria reported 88.2% and 77.0% gain respectively in hearing function in type I tympanoplasty without mastoidectomy (26). Hearing outcome in the present study was affected by ossicular erosion (χ^2^=33.28; P<0.001) as well as mastoid surgery (χ^2^=23.36; P<0.001), but generally there was closure of ABG which increased from 0.8% pre-operatively to 46.7% post-operatively as well as improvement in hearing (with ABG of ≤20dB) from 25.2% pre-operatively to 75.6% post-operatively.

## Conclusion

Tympanoplasty is an effective procedure that can lead to improvement in hearing function of patients and prevention of recurrent ear discharge. Optimal results can be achieved by the use of the appropriate surgical technique. 
